# Draft Genome Sequence of *Komagataeibacter rhaeticus* Strain AF1, a High Producer of Cellulose, Isolated from Kombucha Tea

**DOI:** 10.1128/genomeA.00731-14

**Published:** 2014-07-24

**Authors:** Renato Augusto Corrêa dos Santos, Andresa A. Berretta, Hernane da Silva Barud, Sidney José Lima Ribeiro, Laura Natalia González-García, Tiago Domingues Zucchi, Gustavo H. Goldman, Diego M. Riaño-Pachón

**Affiliations:** a Centro Nacional de Pesquisa em Energia e Materiais (CNPEM), Laboratório Nacional de Ciência e Tecnologia do Bioetanol (CTBE), Campinas, SP, Brazil; b Faculdade de Ciências Farmacêuticas de Ribeirão Preto, Universidade de São Paulo, Ribeirão Preto, SP, Brazil; c Laboratório de Pesquisa, Desenvolvimento e Inovação, Apis Flora Indl. Coml. Ltda., Ribeirão Preto, SP, Brazil; d Instituto de Química, Universidade Estadual Paulista (UNESP), Araraquara, SP, Brazil; e Departamento de Ciencias Biológicas, Universidad de los Andes, Bogotá D.C., Colombia; f Laboratório de Microbiologia Ambiental, EMBRAPA Meio Ambiente, Jaguariúna, SP, Brazil

## Abstract

Here, we present the draft genome sequence of *Komagatabaeicter rhaeticus* strain AF1, which was isolated from Kombucha tea and is capable of producing high levels of cellulose.

## GENOME ANNOUNCEMENT

*Komagataeibacter rhaeticus* AF1, previously known as *Gluconacetobacter rhaeticus* (1), is a Gram-negative rod isolated from Kombucha tea. Briefly, for the isolation of *K. rhaeticus* AF1, 1 ml of Kombucha tea was subjected to serial 10-fold dilutions in 0.85% sterile NaCl solution. Aliquots of each dilution were plated on petri dishes containing Hestrin and Schramm (HS) medium (2). The plates were incubated aerobically at 30°C for 5 days. Suspected colonies were seeded in HS medium and incubated again as described above. After the incubation period, the colonies were transferred to test tubes (20×150 mm) containing HS broth and incubated aerobically at 30°C for five to seven days, in order to evaluate the production of cellulose, which can be easily observed on the surface of the culture medium. The AF1 isolate produced 3.0 g/liter of cellulose.

Here, we present the genome sequence of *Komagataeibacter rhaeticus* strain AF1. This genome was sequenced on the Illumina HiSeq2000 system, generating 44,413,164 paired-end reads of 100 bp (insert size, 250 bp). The reads were preprocessed with the Fastx-Toolkit (http://hannonlab.cshl.edu/fastx_toolkit/), resulting in 43,205,995 paired-end reads. The genome size was estimated to be 4.98 Mbp based on k-mer count statistics (3), with an estimated coverage of 1,561×. Chromosomal assembly was optimized by eliminating reads coming from plasmids, using BLAST (4) and Bowtie (5). The resulting data set was subsampled to a genome coverage of approximately 100×, and this subset was assembled using SPAdes (6, 7). The presence of typical bacterial marker genes was assessed using Amphora2 (8). The remaining reads (raw data set without plasmid-originated reads) were used to extend the contigs and perform scaffolding using SSPACE Basic (9). The Post Assembly Genome Improvement Toolkit was used in order to close gaps and correct substitution and insertion/deletion errors (10). The resulting assembly has 219 scaffolds, with a total length of 3,944,291 bp and an *N*_50_ of 73,183 bp. The average G+C content of the genome is 62.44%, which is similar to those of related species: *K. xylinus* G+C content, 62.1% (11); *K. hansenii* G+C content, 59.5% (12); *K. europaeus* 5P3 G+C content, 61.5%, and *K. oboediens* 174Bp2 G+C content, 61.3% (13); and *Gluconacetobacter diazotrophicus* G+C content, 66.4% (14). Gene prediction was carried out with the NCBI Prokaryotic Genome Annotation Pipeline. A total of 3,460 genes were identified. Of these, 3,358 are protein-encoding genes, and there are 33 pseudogenes, 10 rRNA genes, 58 tRNA genes, and 1 noncoding RNA (ncRNA) gene. The gene content is similar to those of related species, i.e., *K. xylinus* (3,674 genes), *K. hansenii* (3,308 genes), *K. europaeus* 5P3 (3,939 genes), *K. oboediens* 174Bp2 (4,076 genes), and *G. diazotrophicus* (3,472 genes). A search against the UniProt database revealed 3,294 protein-encoding genes with strong sequence similarity hits to proteins in that database. The current genome assembly provides a preliminary landscape of the genomic and metabolic capabilities of *K. rhaeticus*.

### Nucleotide sequence accession numbers.

This whole-genome shotgun project has been deposited at DDBJ/EMBL/GenBank under the accession number JDTI00000000. The version described in this paper is version JDTI01000000.
